# Supplementary figures and images for: Sulforaphane Elicits Protective Effects in Intestinal Ischemia Reperfusion Injury
Source: Int J Mol Sci. 2020 Jul 22;21(15):5189. doi: 10.3390/ijms21155189 (PMC7432940; doi:10.3390/ijms21155189)

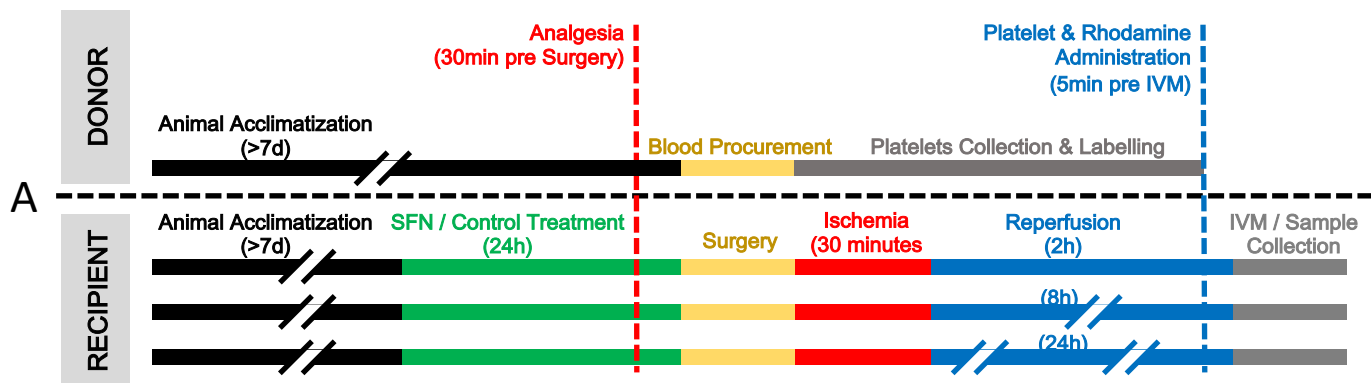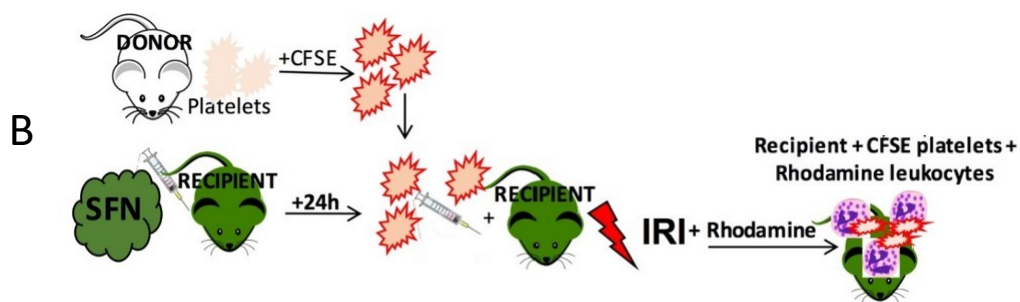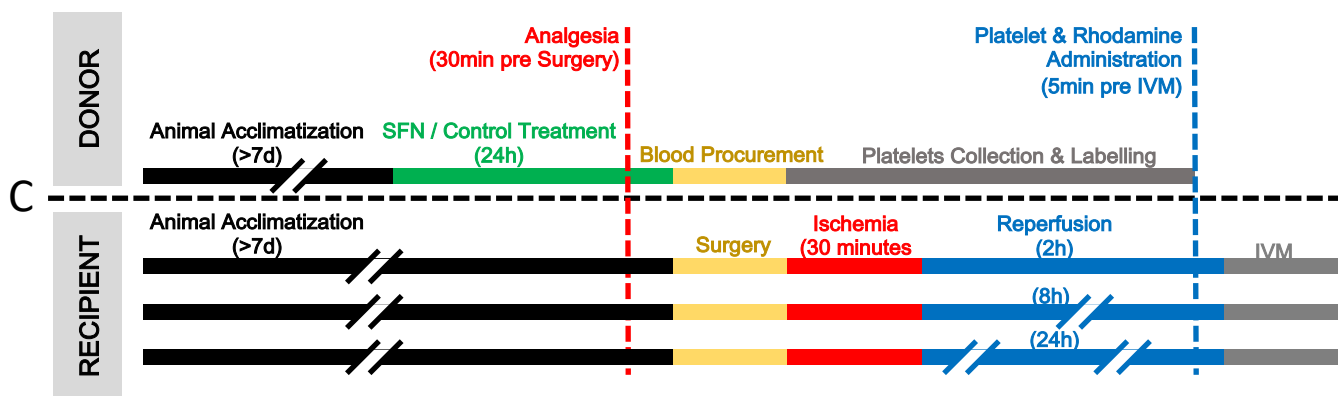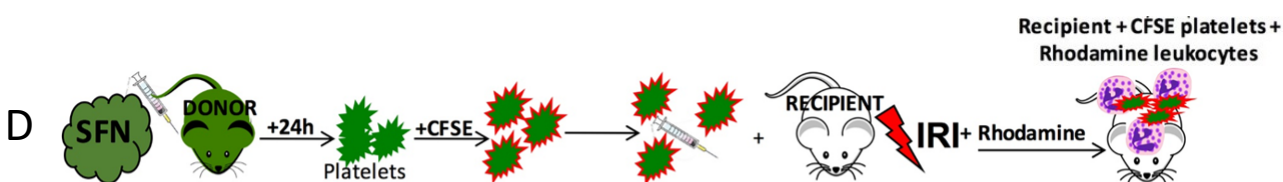

Supplement: Supplementary file 1 [file ijms-21-05189-s001.pdf]
